# Development of Monoclonal Antibody-Based EIA for Tetranor-PGDM which Reflects PGD_2_ Production in the Body

**DOI:** 10.1155/2021/5591115

**Published:** 2021-04-26

**Authors:** Nanae Nagata, Sakura Masuko, Rikako Inoue, Tatsuro Nakamura, Kosuke Aritake, Takahisa Murata

**Affiliations:** ^1^Department of Animal Radiology, Graduate School of Agricultural and Life Sciences, The University of Tokyo, 1-1-1, Yayoi, Bunkyo-ku, Tokyo 113-8657, Japan; ^2^Laboratory of Chemical Pharmacology, Daiichi University of Pharmacy, 22-1 Tamagawa-cho, Minami-ku, Fukuoka 815-8511, Japan

## Abstract

Tetranor-PGDM is a metabolite of PGD_2_. Urinary tetranor-PGDM levels were reported to be increased in some diseases, including food allergy, Duchenne muscular dystrophy, and aspirin-intolerant asthma. In this study, we developed a monoclonal antibody (MAb) and a competitive enzyme immunoassay (EIA) for measuring tetranor-PGDM. Spleen cells isolated from mice immunized with tetranor-PGDM were utilized to generate Ab-producing hybridomas. We chose hybridomas and purified MAb against tetranor-PGDM to develop competitive EIA. The assay evaluated the optimal ionic strength, pH, precision, and reliability. Specificity was determined by cross-reactivity to tetranor-PGEM, tetranor-PGFM, and tetranor-PGAM. Recovery was determined by spiking experiments on artificial urine. Optimal ionic strength was 150 mM NaCl, and optimal pH was pH 7.5. Metabolites other than tetranor-PGDM did not show any significant cross-reactivity in the EIA. The assay exhibited a half-maximal inhibition concentration (IC_50_) of 1.79 ng/mL, limit of detection (LOD) of 0.0498 ng/mL, and range of quantitation (ROQ) value of 0.252 to 20.2 ng/mL. The intra- and inter-assay variation for tetranor-PGDM was 3.9–6.0% and 5.7–10.4%, respectively. The linearity-dilution effect showed excellent linearity under dilution when artificial urine samples were applied to solid-phase extraction (SPE). After SPE, recovery of tetranor-PGDM in artificial urine averaged from 82.3% to 113.5% and was within acceptable limits (80%–120%). We successfully generated one monoclonal antibody and developed a sensitive competitive EIA. The established EIA would be useful for routine detection and monitoring of tetranor-PGDM in research or diagnostic body fluids.

## 1. Introduction

Tetranor-PGDM is a metabolite of PGD_2_ and reflects the biosynthesis of PGD_2_ in mice and humans [1]. We previously reported that the urinary levels of tetranor-PGDM reflect the mast cell activity and severity of symptoms in patients with food allergies [2, 3]. Other studies also reported that urinary tetranor-PGDM levels were elevated in other diseases, such as Duchenne muscular dystrophy [4] or aspirin-intolerant asthma patients [5]. Therefore, this urinary index improves the diagnosis and therapeutic procedure against these diseases.

Currently, the measurement of urinary tetranor-PGDM requires liquid chromatography tandem mass spectrometry (LC-MS/MS). These instrumental analyses are expensive and require highly trained experts. When clinical use of urinary tetranor-PGDM measurements is considered, the use of EIA would be suitable because of its ease, convenience, and quickness. Although the anti-tetranor-PGDM serum-based competitive enzyme immunoassay (EIA) kit is commercially available, the system utilizes a polyclonal antibody and is applicable only for research. A monoclonal antibody-based EIA system is required to expand the diagnostic system using urinary tetranor-PGDM as an index.

The aim of the present study was to generate specific monoclonal antibodies against tetranor-PGDM and develop a competitive EIA to measure the tetranor-PGDM level in body fluids.

## 2. Materials and Methods

### 2.1. Mice

Six-week-old female BALB/c mice were obtained from CLEA Japan (Tokyo, Japan). They had free access to a standard diet (MF; Oriental Yeast Co., Tokyo, Japan) and water. All procedures and animal care were approved by the Committee on the Ethics of Animal Experiments, Graduate School of Agriculture and Life Sciences, Tokyo University, and conducted according to the Guidelines for Animal Experiments of the Graduate School of Agriculture and Life Sciences, Tokyo University (approval no: P18-069).

### 2.2. Preparation of Monoclonal Antibody

BALB/c mice were subcutaneously immunized with keyhole limpet hemocyanin- (KLH-) conjugated tetranor-PGDM. Splenocytes from the immunized mice were fused with myeloma cells (SP2/0-Ag14) under standard protocols [6]. Positive hybridomas were cloned using the limiting dilution method. IgG antibody was purified from the serum-free medium of hybridoma cultures.

### 2.3. Competitive Enzyme-Linked Immunoassay

The reactivity of the tetranor-PGDM monoclonal antibody was determined by competitive EIA. A precoated (Goat Anti-Mouse IgG) EIA 96-well strip plate (Cayman Chemical, Michigan, USA) was treated with various concentrations of tetranor-PGDM monoclonal antibody for 1 h. The plate was then washed three times with phosphate-buffered saline (PBS) containing 0.5% bovine serum albumin (BSA) and 0.05% (*v*/*v*) Tween-20 (PBS-T). Subsequently, tetranor-PGDM-linked acetylcholinesterase tracer (tracer) was incubated overnight. The plate was then washed three times with PBS-T and incubated with Ellman's reagent for 1 h. Absorbance was measured at 405 nm with a microplate reader (Perkin Elmer, MA, USA). Selectivity of tetranor-PGDM against various prostaglandin metabolites was analyzed by competitive EIA using tetranor-PGEM, tetranor-PGAM, and tetranor-PGFM.

### 2.4. EIA Establishment

The effects of pH (5.0, 6.0, 6.5, 7.0, 7.5, 8.0, and 9.0) and ionic strength (NaCl content in 10 mM phosphate buffer) on EIA performance were tested. Under each condition, a standard inhibition curve for tetranor-PGDM was plotted. Cross-reactivity values were calculated based on the following formula: cross‐reactivity (%) = (IC_50_ tetranor‐PGDM/IC_50_ other tetranor‐PGM) × 100.

### 2.5. Recovery Tests

The spiked tetranor-PGDM into the assay solution was analyzed using the proposed EIA. Interday variation was determined based on nine replicates, and intraday variation was measured on three consecutive days.

### 2.6. Theoretical Error

The data sets of the analyte concentration and absorbance were fitted with the following four-parameter logistic equation containing the fitting parameters of *a*, *b*, *c*, and *d* [7, 8]:

(1) $$\begin{array}{c} f\left(X\right)=\frac{a–d}{1+{\left(X/c\right)}^{b}}+d. \end{array}$$

Based on a model proposed by Hayashi et al. [9], the relative standard deviation (RSD) in the entire analysis (*ρ*_*T*_) is expressed as

(2) $$\begin{array}{c} {\rho_{T}}^{2}=\frac{X^{2}}{{\left(X+G\right)}^{2}}\left({\rho_{X}}^{2}+{\rho_{G}}^{2}\right)+{\rho_{B}}^{2}+{\rho_{S}}^{2}+{\left(\frac{\sigma_{W}}{f\left(X\right)}\times 100\right)}^{2}. \end{array}$$

In this formula, *G* is the amount of labeled antigen. *ρ*_*X*_, *ρ*_*G*_, *ρ*_*B*_, and *ρ*_*S*_ denote the RSDs of the pipetted volumes of the analyte, labeled antigen, antibody, and substrate, respectively. *ρ*_*S*_ denotes two-thirds of the RSD of the pipetted volumes of the chromogen substrate solution. *σ*_*W*_ denotes the SD of the inherent absorbances between the wells in a plate. However, we could not determine the concentration of labeled antigen; therefore, we used the approximate equation of uncertainty for the competitive EIA:

(3) $$\begin{array}{c} {\rho_{T}}^{2}={\rho_{B}}^{2}+{\left(\frac{\sigma_{W}}{f\left(X\right)}\times 100\right)}^{2}. \end{array}$$

The values employed were *ρ*_*B*_ = 0.782 and *σ*_*W*_ = 0.0026. *ρ*_*T*_ was calculated from blank-subtracted measurements. To determine the values of *ρ*_*B*_, the weight of the pipetted solution was measured 20 times and the RSD was calculated. The value of *σ*_*W*_ was obtained from the between-well SD of absorbances for empty wells (*n* = 96).

### 2.7. Measurement of Tetranor-PGDM in Artificial Urine

Artificial urine (Supplementary Table 1) was spiked with tetranor-PGDM in distilled water at four levels (1.25, 2.5, 5.0, and 10.0 ng/mL). For solid-phase extraction (SPE), 0.4 mL of the urine sample was diluted to 0.8 mL with 0.1% (*v*/*v*) formic acid. The mixed solutions were applied to an SPE cartridge (HLB *μ*Elution plate, Waters, Massachusetts, USA) preconditioned with 200 *μ*L acetonitrile and distilled water. After washing with 200 *μ*L distilled water and 200 *μ*L hexane, the lipid fractions were eluted with 50 *μ*L acetonitrile. The eluate was collected and dried *in vacuo*. The resulting residue was reconstituted in 0.4 mL of assay solution. The sample solution containing tetranor-PGDM was then introduced into the EIA. At the same time, tetranor-PGDM was measured by LC-MS/MS as previously described [2, 3]. The recovery from the SPE procedure was found to be 77.1% by LC-MS/MS (Supplementary Table 2).

## 3. Results

### 3.1. Optimization of Competitive EIA

**Figure 1:**
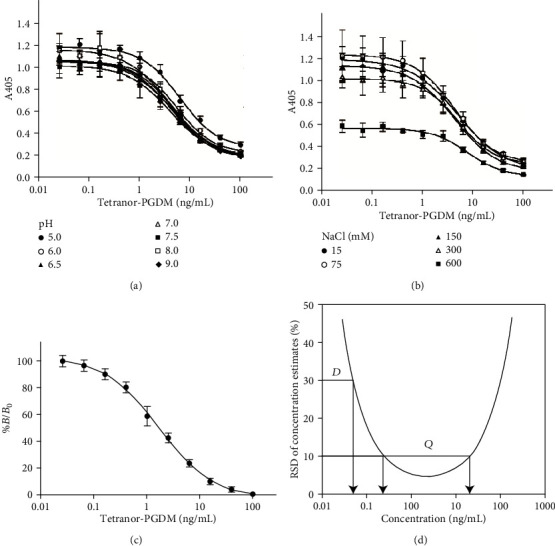
Optimization of competitive EIA. Effects of pH value (a) and ionic strength (b) on assay buffer on performance of EIA. (c) Inhibition curve for tetranor-PGDM under the optimized conditions. (d) Detection limit and quantitation range for tetranor-PGDM. Arrow *D*: the detection limit; arrow *Q*: the lower and upper limits of quantitation range.

We obtained hybridomas that produced MAb against tetranor-PGDM from immunized mouse spleen cells. Then, MAb was purified and applied to competitive EIA. As each urine sample from patients has different physicochemical features, we examined the optimal ion strength and pH for the MAb-based EIA. As shown in Figure 1(a), the 600 mM NaCl concentration strongly interferes with the competitive curve. IC_50_ values of 6.6, 5.7, 5.3, and 7.3 ng/mL were obtained with NaCl concentrations of 15, 75, 150, and 300 mM in the assay buffer, respectively. Therefore, the optimal salt concentration was set to 150 mM NaCl in phosphate buffer. To determine the optimal pH of the assay buffer, IC_50_ values of 8.6, 6.6, 5.5, 5.6, 4.8, 5.2, and 4.8 ng/mL were obtained with pH values 5.0, 6.0, 6.5, 7.0, 7.5, 8.0, and 9.0, respectively (Figure 1(b)). These results indicated that the assay was more sensitive under neutral to slightly alkaline conditions than under slightly acidic conditions. Thus, the optimal pH of the assay buffer was set at 7.5. Under the optimized conditions, a competitive curve for tetranor-PGDM was established with an IC_50_ value of 1.79 ± 0.36 ng/mL (Figure 1(c)).

### 3.2. Validation of Generated EIA

We next evaluated the precision and reliability. Tetranor-PGDM was serially diluted in assay solution and used to test the detection limit of tetranor-PGDM. The limit of detection (LOD) was estimated to be 0.0498 ng/mL, and the range of quantitation (ROQ) value was estimated from 0.252 to 20.2 ng/mL. The LOD and ROQ are defined as the concentration with 30% RSD and region with <10% RSD, respectively (Figure 1(d)). The imprecision in this EIA was assessed using an assay solution containing tetranor-PGDM at three different concentrations. Intra- and inter-assay CVs ranged from 3.9% to 6.0% (*n* = 9) and from 5.7% to 10.4% (*n* = 9), respectively (Table 1). The recovery of tetranor-PGDM ranged from 89.4% to 111.7%. All recoveries and coefficients of variation of tetranor-PGDM were acceptable.

### 3.3. Cross-Reactivity of Generated MAb against Tetranor-PGDM

**Figure 2:**
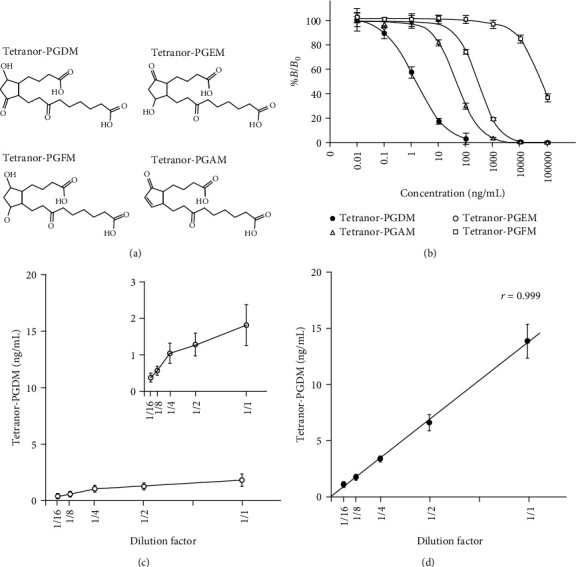
Analysis of tetranor-PGDM in urine. (a) Lipids for cross-reactivity test of tetranor-PGDM. (b) Inhibition curves for tetranor-PGDM, tetranor-PGEM, tetranor-PGAM, and tetranor-PGFM. Linearity of dilution curves for artificial urine samples before (c) and after (d) solid-phase extraction. The artificial urine sample with a high tetranor-PGDM level was diluted stepwise with the assay solution.

In addition to tetranor-PGDM, other urinary PG metabolites, such as tetranor-PGEM and tetranor-PGFM, are present in urine (Figure 2(a)). We next assessed the cross-reactivity of tetranor-PGEM, tetranor-PGAM, and tetranor-PGFM. As shown in Figure 2(b), the closed cycle indicates the typical competitive curve of tetranor-PGDM. The competitive curves against other urinary PG metabolites were shifted to the right. Cross-reactivities with tetranor-PGEM, PGAM, and PGFM were 0.631%, 3.876%, and 0.003%, respectively (Supplementary Table 3). Thus, these metabolites did not exhibit any significant cross-reactivity with the EIA, suggesting that the present EIA system can measure urinary tetranor-PGDM with sufficient specificity.

### 3.4. Analysis of Tetranor-PGDM in Artificial Urine

The linearity dilution effect is an indicator of the validity of the proposed method. Finally, artificial urine samples containing 20 ng/mL tetranor-PGDM were serially diluted and assayed. The concentration of tetranor-PGDM detected did not fit a linear correlation with the dilution ratios. In addition, the recoveries of tetranor-PGDM were less than 30.2% (Figure 2(c)). These results suggested that some urine components could cause negative interference in the performance of the EIA system. Thus, urine samples were applied to the SPE. The recovery of tetranor-PGDM from the SPE procedure was found to be 77.1% by LC-MS/MS analysis (Supplementary Table 2). After SPE, artificial urine samples containing 13.9 ng/mL tetranor-PGDM were serially diluted and assayed. The dilution curves of artificial urine after SPE featured robust linearity (*r* = 0.999, Figure 2(d)).

To determine recovery, we used artificial urines with different concentrations of tetranor-PGDM. The recovery of tetranor-PGDM ranged from 82.3% to 113.5%. All recoveries of tetranor-PGDM were acceptable (Table 2).

## 4. Discussion

In this study, we generated an anti-tetranor-PGDM monoclonal antibody and a competitive EIA for the measurement of tetranor-PGDM. Our EIA proved to be sensitive enough to allow quantification of tetranor-PGDM from 0.252 to 20.2 ng/mL. Furthermore, we confirmed the accuracy of the quantified values using spiked samples with known amounts of tetranor-PGDM. Strong average recoveries (89.4% to 111.7%) with a coefficient of variation from 3.9% to 10.4% were obtained for the assay solution samples.

As antigen-antibody binding is characterized by weak intermolecular bonds, a change in either ionic strength or pH could affect this interaction. The individual features of each urine sample may affect the reliability of the analysis using EIA methods [10]. The IC_50_ value of EIA did not change markedly up to pH 9.0. Increasing the ionic strength up to 300 mM did not markedly change the recognition of tetranor-PGDM. These results suggest that a change in either ionic strength or pH has little effect on the EIA using this MAb. Since urine samples are easy to collect in clinical settings, we assumed the use in urine and evaluated the accuracy and reliability using artificial urine samples. The matrix effect of the samples was eliminated using SPE. After SPE, robust average recoveries (82.3% to 113.5%) were obtained for urine samples. Therefore, our EIA has the potential to be applied to media from a variety of cultured cells and tissues and body fluids such as plasma and urine from animals and humans.

In healthy individuals, the urinary tetranor-PGDM concentration is 1.5 ± 0.3 ng/mg creatinine (Cre) [1]. The urinary Cre concentration was approximately 1 mg/mL [11]. We previously showed that the optimal cut-off value for urinary tetranor-PGDM during oral food challenge of food allergic patients was 2.25 ng/mg Cre [3]. The LOD value was lower than the cut-off value. In another disease, the urinary tetranor-PGDM concentration was reported to be 9.7 ng/mg Cre in Duchenne muscular dystrophy patients [4]. These results showed that this optimized EIA method can be acceptable for detection of urinary tetranor-PGDM in patients with several diseases.

The urinary concentrations of tetranor-PGEM and tetranor-PGFM were 8–15 ng/mg Cre and 11–59 ng/mL, respectively [12, 13]. Our competitive EIA showed negligible cross-reactivity with other tetranor-PG metabolites. The KLH-conjugated tetranor-PGDM, prepared by conjugating KLH on the carboxyl groups of tetranor-PGDM via NHS/EDC-mediated esterification, was utilized to immunize mice. Generally, antibodies are thought to best recognize the part of the hapten that is most distant from conjugate linkage. Thus, the epitopes for MAb are thought to be in the cyclopentane ring of tetranor-PGDM. In addition, the MAb did not recognize tetranor-PGEM, tetranor-PGFM, or tetranor-PGAM, which do not have the 11-keto group. This suggests that the epitope for MAb was in the 11-keto tetranor-PGDM group. Taken together, cross-reactivity experiments showed that MAb was directed almost exclusively against the tetranor-PGDM structure.

## 5. Conclusions

In this study, we successfully generated one monoclonal antibody and developed a sensitive competitive EIA. This EIA method is useful for the quantification of the tetranor-PGDM in body fluids, for evaluating animal disease models, and for the index of diagnosis and therapeutic monitoring of food allergy or other diseases.

## Figures and Tables

**Table 1:** Precision of the proposed EIA for tetranor-PGDM measurement at different concentration levels.

Concentration (ng/mL)					Mean recoveries (%)
Spiked	Mean		SD	CV (%)	
Within-run (*n* = 9)					
1.024	1.14	±	0.07	6.0	111.7
2.56	2.29	±	0.11	4.8	89.4
6.4	6.19	±	0.24	3.9	96.6
Between-day (*n* = 9)					
1.024	1.10	±	0.11	10.4	107.6
2.56	2.45	±	0.14	5.7	95.5
6.4	6.25	±	0.41	6.6	97.7

**Table 2:** Analytical recovery of tetranor-PGDM in artificial urine samples with different tetranor-PGDM concentrations.

Concentration (ng/mL)				Mean recoveries (%)
Sample	Spiked	Theoretical	Measured	
AU-1 (*n* = 3)	0	—	0.85	—
	0.86	1.32	1.49	113.5
	1.87	2.10	2.09	100.0
	3.91	3.67	3.81	103.8
	9.12	7.68	7.04	93.0
AU-2 (*n* = 3)	0	—	5.95	—
	0.86	5.25	5.53	105.6
	1.87	6.03	6.53	108.4
	3.91	7.60	6.99	91.9
	9.12	11.62	9.53	82.3

Concentration of tetranor-PGDM was 0.85 (AU-1) and 5.95 (AU-2). The theoretical concentrations were corrected using the recovery value from the SPE procedure (77.1% by LC-MS/MS).

## Data Availability

All data were shown in the manuscript.

## Supplementary Materials

Supplementary Materials Supplementary Table 1: the composition of artificial urine. Supplementary Table 2: recovery from SPE. Supplementary Table 3: cross-reactivity among tetranor-PGDM and related lipids.
