# Supplementary material for: Development of Monoclonal Antibody-Based EIA for Tetranor-PGDM which Reflects PGD2 Production in the Body
Source: J Immunol Res. 2021 Apr 26;2021:5591115. doi: 10.1155/2021/5591115 (PMC8096570; doi:10.1155/2021/5591115)
Supplement: Supplementary Materials — Supplementary Table 1: the composition of artificial urine. Supplementary Table 2: recovery from SPE. Supplementary Table 3: cross-reactivity among tetranor-PGDM and related lipids. [file 5591115.f1.docx]

**Supplementary Table 1. The composition of artificial urine**

|  | mM |
| --- | --- |
| Na_2_SO_4_ | 11.965 |
| C_5_H_4_N_4_O_3_ | 1.487 |
| Na_3_C_6_H_5_O_7_.2H_2_O | 2.450 |
| C_4_H_7_N_3_O | 7.791 |
| CH_4_N_2_O | 249.750 |
| KCl | 30.953 |
| NaCl | 30.053 |
| CaCl_2_ | 1.663 |
| NH_4_Cl | 23.667 |
| K_2_C_2_O_4_.H_2_O | 0.19 |
| MgSO_4_.7H_2_O | 4.389 |
| NaH_2_PO_4_.2H_2_O | 18.667 |
| Na_2_HPO_4_.2H_2_O | 4.667 |

**Supplementary Table 2. Recovery from SPE.**

|  | Area | | Recovery  (%) | Mean Recovery  (%) |
| --- | --- | --- | --- | --- |
| Sample | SPE | |  |  |
|  | - | + |  |  |
| 1 | 9228 | 7844 | 85.0 | 77.1 |
| 2 | 10937 | 7970 | 72.9 |  |
| 3 | 10215 | 7508 | 73.5 |  |

**Supplementary Table 3. Cross-reactivity among tetranor-PGDM and related lipids.**

| Lipid | Cross-reactivity  (%) |
| --- | --- |
| tetranor-PGDM | 100.0 |
| tetranor-PGEM | 0.631 |
| tetranor-PGAM | 3.876 |
| tetranor-PGFM | 0.003 |
